# A Genetic Test to Identify People at High Risk of Heart Failure

**DOI:** 10.3390/ijms26041782

**Published:** 2025-02-19

**Authors:** Xintian Ge, Bek Brittain, Luke Dawson, Girish Dwivedi, David M. Kaye, Grant Morahan

**Affiliations:** 1 Centre for Diabetes Research, Harry Perkins Institute of Medical Research, Nedlands, WA 6009, Australia; cynthia.ge@uwa.edu.au (X.G.); bek.brittain@perkins.uwa.edu.au (B.B.); girish.dwivedi@perkins.uwa.edu.au (G.D.); 2 Stroke Research Centre, Perron Institute for Neurological and Translational Science, Nedlands, WA 6009, Australia; 3 Heart Failure Research Group, Baker Heart and Diabetes Institute, Melbourne, VIC 3004, Australia; luke.dawson1@monash.edu (L.D.); david.kaye@baker.edu.au (D.M.K.); 4 Department of Cardiology, Fiona Stanley Hospital, Perth, WA 6150, Australia; 5 Advanced Genetic Diagnostics, Nedlands, WA 6009, Australia

**Keywords:** heart failure, genetic risk, personalized risk score, precision medicine, complex genetic diseases

## Abstract

Earlier intervention may delay or prevent heart failure (HF), a widespread health problem. However, it is not currently possible to identify those who are most at risk, especially before the appearance of any clinical signs. This study presents the development and subsequent validation of a novel genetic test for predicting the risk of HF, utilizing data from three independent cohorts of Australian and US subjects. We developed a first-phase test using the Baker Biobank case–control cohort, identifying 41 genetic variants indicative of HF risk through genome-wide interaction and association analyses. Subsequently, a second-phase test was designed. This identified 29 additional single-nucleotide polymorphisms. The combination of these two tests resulted in an aggregate test with a high predictive accuracy, achieving an Area Under the Curve of 0.93 and a balanced accuracy of 0.89. High genetic risk subjects in the Baker Biobank cohort had an odds ratio of 533.2. The test’s robustness was validated by applying it to data from the Busselton Health Study and the Atherosclerosis Risk in Communities cohorts, yielding, respectively, Areas Under the Curve of 0.83 and 0.72, a balanced accuracy of 0.76 and 0.67, and Odds Ratios of 12.3 and 4.6. These results highlight the critical role of genetic factors in the development of heart failure and demonstrate this test’s potential as a significant tool for clinical HF risk prediction.

## 1. Introduction

Heart failure (HF) is a critical global health issue affecting over 26 million people and has increasing prevalence in aging populations [1]. In the USA, it has been estimated that over 8 million people will have HF by 2030, and its real total direct medical costs are projected to increase (in USD as of 2010) from USD 21 billion to USD 53 billion [2]. HF is a complex syndrome, characterized by the heart’s inability to pump or adequately fill with blood, manifesting in two primary forms: systolic and diastolic heart failure [3,4]. Systolic heart failure is marked by a reduced ejection fraction and occurs when the heart muscle weakens and fails to contract effectively, often due to damage from conditions like myocardial infarction (MI) [5]. Diastolic heart failure, in contrast, involves the heart becoming stiff and unable to fill properly despite a normal ejection fraction, commonly associated with hypertension and age-related changes in the heart muscle [6].

The pathophysiology of HF is complex, involving an interplay of hemodynamic stress, neurohormonal imbalances, and cellular alterations in the heart [7]. Structural changes such as cardiac hypertrophy and dilation, together with cellular responses like apoptosis and fibrosis, play significant roles. The HF risk factors are diverse, including hypertension, which places increased strain on the heart; coronary artery disease (CAD), leading to impaired heart muscle function; and diabetes, which independently exacerbates HF risk. Lifestyle factors like smoking, obesity, and a poor diet also contribute significantly [8,9].

There is a need for primary prevention and earlier treatment of at-risk individuals. The performance of population risk equations for HF, such as the PC equations to Prevent HF (PCP-HF), is only moderate and largely reliant on age rather than other risk factors [10]. This limitation highlights the need for earlier intervention to delay or prevent HF, but it is challenging to identify those who are most at risk, especially before the appearance of any clinical signs [11]. Diagnosing HF presents significant challenges due to its clinical complexity and the variability in its symptoms and disease progression [3,12]. Patients with HF may exhibit a wide range of symptoms, from fatigue and dyspnea to edema and weight gain, which can overlap with other cardiac and non-cardiac conditions, complicating am accurate diagnosis [13]. The current diagnostic tools, while essential, have limitations [13]. Echocardiography, the cornerstone of HF diagnosis, primarily assesses the structural and functional aspects of the heart but may not detect early or subtle forms of the disease [14]. Similarly, while natriuretic peptides like BNP and NT-proBNP are valuable in diagnosing HF, their levels can be influenced by other factors such as age, renal function, and obesity, potentially leading to diagnostic ambiguity [15]. The need for more precise diagnostic methods is evident, as a misdiagnosis or delayed diagnosis of HF can have serious consequences. Inaccurate or late identification of HF can lead to inappropriate or delayed treatment, resulting in worsened patient outcomes, an increased risk of hospitalization, the progression of the disease, and even a heightened mortality risk [16]. Enhancing the accuracy and speed of diagnosis is vital for effective HF management, highlighting the need for advancements in the diagnostic technologies and strategies within the field of heart failure [17].

Genetic testing offers the advantage of detecting individuals at a higher risk of HF years before the development of any clinical signs [18]. However, no suitable genetic test for HF risk is currently available [19]. To address this gap, we aimed to develop a genetic test for HF risk using three independent cohorts established using rigorous diagnostic criteria. In this paper, we describe the development, performance, and validation of a genetic test that identifies individuals at a higher risk of HF.

## 2. Results

### 2.1. The First-Phase HF Test

Our goal was to develop a genetic test that could distinguish between HF cases and controls using the Baker Biobank (BB) cohort. The controls were individuals over the age of 70 without HF. We divided the dataset into “discovery” and “testing” subsets. We began by partitioning the training set into two groups and selected 23 single-nucleotide polymorphism (SNP)s that showed the most significant associations in our studies. We also identified 41 SNPs as having significant interactions (Supplementary Table S2). Next, we implemented a multi-stage deep neural network (DNN) pipeline that leveraged weight adjustments from previous training cycles for enhanced accuracy. The genotypes at the identified SNPs were integrated into the DNN to generate a model to distinguish effectively between cases and controls.

This method generated a Quantitative Risk Score (QRS) for each individual. We identified the optimal percentile of the control group to set as the threshold for risk definition (Supplementary Figure S1). The optimum threshold was set at the 90th percentile of the control values (that is, only 10% of the controls had a higher QRS than this value). High-risk subjects were thus defined based on having a QRS that exceeded the 90th percentile of the controls (i.e., a QRS = 0.402). Subjects below this score were considered to be low-risk. As shown in Table 1, the model yielded an odds ratio (OR) of 2.53 and a hazard ratio (HR) of 5.7, a substantial improvement over the traditional polygenic risk scores (PRSs) for heart disease, which typically exhibit ORs below 2. This demonstrates the value of our model in identifying individuals at high risk for HF and indicates its potential as a valuable tool in preventive cardiology.

As shown in Table 1, this test was effective in identifying cases, with ~72% of the cases identified as high-risk. However, the test was not good at identifying the controls, i.e., subjects who were not diagnosed with HF. There are at least two possible reasons for this. The first is that these high-risk people may develop HF at a later age. The second explanation is that HF may arise due to more than one molecular mechanism, with each affected by separate genetic variants. To account for such genetic heterogeneity, we developed a second-phase test.

### 2.2. A Second-Phase Genetic Test for HF Risk

We masked all 41 SNPs from the genome build to perform association studies on the subjects defined as high-risk by Test 1. Utilizing the previously established methodology from Test 1, we identified a total of 29 SNPs to form the basis of Test 2. The threshold for Test 2 was determined as the 90th percentile of the controls (at a QRS value of 0.541). Using this threshold, we evaluated the performance of Test 2 on the reserved “test set” of BB subjects defined as high-risk by Test 1 (Table 2).

### 2.3. Combining the Two Genetic HF Risk Tests

**Figure 1:**
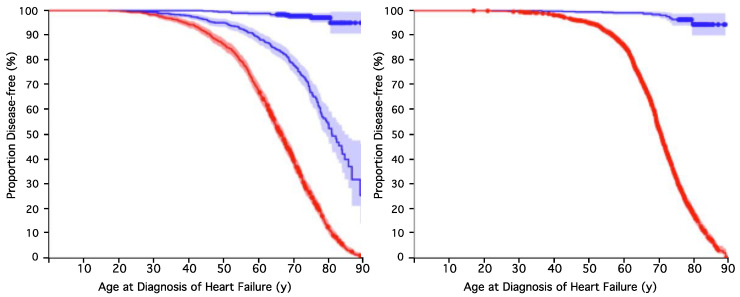
Kaplan–Meier (KM) survival curves of genetic HF risk test. Results are shown for the “test set” of the BB cohort (i.e., the subjects whose data were not considered in the test development phase). Risk groups were assigned on the basis of genetic data only: no clinical data were used in assigning risk. (**Left panel**): The red line represents the predicted high-risk group, and blue lines represent the intermediate- and low-risk groups. Shaded areas indicate 95% confidence intervals (CI). (**Right panel**): Combined HF test merging intermediate- and high-risk groups, yielding an AUC of 0.93, a BAC of 0.89, and an OR = 123.1, with *p* = 5.6 × 10^−222^ The HR of the combined test in the entire BB cohort was 157.6.

The two tests were designed to be complementary. As each defines a different aspect of genetic heterogeneity in HF, we can combine the two tests to predict the overall genetic risk of HF. Combining the results for both HF tests yielded four distinct subsets of groups as follows: high-risk in both tests (1_1); high-risk in the first test and low-risk in the second test (1_0); low-risk in the first test and high-risk in the second test (0_1); and low-risk in both tests (0_0). These four combinations can be assigned to three levels of risk, with the 1_1 and 0_0 groups considered high- and low-risk, respectively. The remaining two groups, 0_1 and 1_0, were represented as intermediate-risk. The Kaplan–Meier (KM) survival graph and the 2 × 2 contingency table for the combined HF test are shown in Figure 1 and Table 3. The separation between the survival curves was highly significant, indicating that the risk assignment of HF after both tests was biologically meaningful. There were considerably more cases in the high- and intermediate-risk groups (i.e., 1_1, 1_0, and 0_1) than those in the low-risk group (0_0). The right-hand panel in Figure 1 shows the result after merging the intermediate- and high-risk groups.

The performance of the combined test is indicated by the *p*-value from Fisher’s exact test and the OR scores (Table 3). The combined test identifies people at high genetic risk whose odds of having heart failure are over forty times that of those at low risk. Over 90% of the subjects identified as being at high genetic risk were diagnosed with HF before they reached 80 years of age. In contrast, less than 5% of the subjects that we predicted as low-risk ever developed HF. The intermediate-risk group also had a much higher HF incidence than those in the low-risk group: about 50% developed heart failure by 80 years of age.

### 2.4. Validation of the Genetic HF Risk Tests

**Figure 2:**
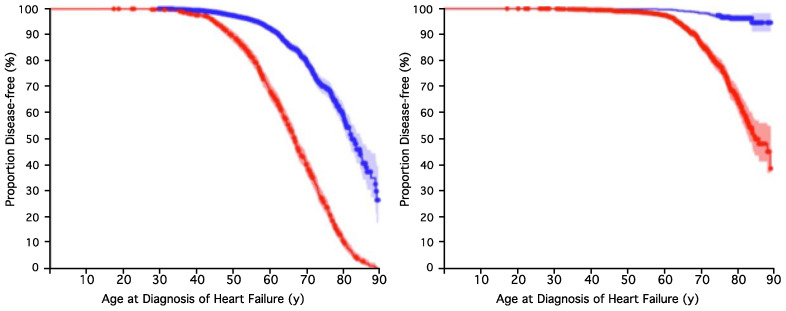
Validation of the combined genetic HF risk tests in the ARIC (**left panel**) and BHS (**right**) cohorts, with *p* = 2.3 × 10^−57^ and *p* = 1.2 × 10^−70^ for ARIC and BHS, respectively. Results are shown with the intermediate- and high-risk groups merged. The red line represents the predicted high-risk group, and blue lines represent the intermediate- and low-risk groups. Shaded areas indicate 95% confidence intervals.

In order to confirm the genetic risk tests, they were applied to data from two independent population-based cohorts, the Atherosclerosis Risk in Communities (ARIC) heart failure cohort and the Busselton Health Study (BHS). Please note that this study design meant that the number of cases comprised a much lower proportion than that in the BB case–control cohort. The results of the prediction of Test 1 for both cohorts are shown in Table 4. These indicate very significant results with an OR > 4 in the BHS cohorts. Test 2 was also validated in these cohorts. Figure 2 and Table 5 show the results of the combined tests (with the intermediate- and high-risk groups merged). The Relative Risk (RR) in the ARIC cohort was 2.6, while the RR in the BHS was 10.5, indicating that people at a higher genetic risk were over ten times more likely to develop HF than those at low genetic risk in the BHS.

### 2.5. Comparison with Risk Prediction Based on Clinical Factors

Among the 1604 individuals in the BB cohort with no prior history of HF at their entry to the study, 30 commonly used clinical variables (including age, sex, socioeconomic status, alcohol consumption, waist circumference, body mass index (BMI), systolic and diastolic blood pressure (BP), blood levels of lipids (HDL and LDL), triglycerides, glucose and cholesterol, smoking status, co-morbidities (e.g., stroke, diabetes, MI, hypertension, etc.), cholesterol-lowering medications, BP medications, and diabetes medications) were included as predictors in a Cox regression model for incident HF over a mean 10.1-year follow-up. The C-statistic for the prediction model incorporating these variables was 0.75. In contrast, the genetic test had a C-statistic of 0.804. Heart failure commonly affects older men, and adding age and sex traits raised the score to 0.835. Adding in all of the remaining clinical variables increased the C-score only marginally, to 0.86 (a difference of 0.11 in the C-statistic with the genetic test and clinical variables compared to that with the clinical variables alone).

## 3. Discussion

The development and validation of a novel genetic test for predicting HF risk, utilizing data from three independent cohorts, represent a significant advancement in cardiovascular genetics and personalized medicine, offering the potential for early risk stratification and intervention. Our approach, which involved creating two independent risk tests, also provides valuable insights into the complex genetic architecture of HF.

The first-phase test, comprising 41 genetic variants, demonstrated a superior performance compared to that of the conventional polygenic risk scores (PRSs) for cardiovascular diseases. While PRSs for conditions such as MI typically yield hazard ratios below 1.5 [20], our test achieved a significantly higher predictive accuracy. This underscores the potential of our approach to uncover robust genetic associations with HF. The success of this test aligns with emerging evidence that HF risk is influenced by a combination of common and rare genetic variants, as well as interactions with environmental factors [21,22].

The complementary second test focused on refining the risk prediction in the high-risk individuals identified by the first test, revealing the influence of an additional 29 SNPs. This two-phase approach addressed the genetic heterogeneity of HF and significantly improved the predictive accuracy, achieving an area under the curve (AUC) of 0.93 and a balanced accuracy (BAC) of 0.89 in the BB cohort. This innovative strategy was inspired by our earlier studies showing that stratification of the patient groups increases the evidence of genetic influences [23,24] and highlights the importance of iterative refinement in genetic risk prediction. By integrating advanced machine learning techniques to optimize the risk stratification, this test sets a new benchmark for genetic prediction of cardiovascular disease.

Our genetic test was validated in independent cohorts recruited from different continents, namely the ARIC and BHS cohorts. This demonstrates the consistent performance of the test across diverse populations. Notably, the test was also significant in African-American participants from the ARIC cohort (Supplementary Table S3), suggesting that the test may have broad applicability across ethnic groups. However, the variability in its performance across cohorts underscores the need for further refinement in non-European populations. This is consistent with findings from other genetic studies, which have highlighted differences in the allele frequencies and linkage disequilibrium patterns across ethnic groups [25]. Future research should aim to identify population-specific variants and refine the test to improve its accuracy in diverse populations.

Some possible limitations of our study warrant discussion. The BB cohort was predominantly of European descent (73%), with the remaining 27% representing many diverse ethnicities. This may limit the generalizability, as the genetic risk varies across ethnic groups, and population stratification, despite principal component analysis (PCA) adjustments, remains a potential confounding variable that affects genetic associations. While the test performed well in African-American participants from the ARIC cohort, broader validation is needed. Additionally, age and sex differences between the cases and controls may have introduced bias, as HF risk increases with age and is more prevalent in men. This was reflected in differences in the makeup of the controls and cases in the BB cohort, with males comprising 47% of the controls and 53% of the cases. Despite this potential bias, our risk test identified women who were diagnosed with HF. Missing genotype data required imputation in the ARIC and BHS cohorts, which may have introduced inaccuracies that reduced the test’s performance.

The diagnoses of HF were based on the International Classification of Diseases (ICD) coding conducted individually within each cohort. Whilst this introduces the potential for some variability, prior studies have shown that the ICD codes for HF are generally robust [26]. Furthermore, the large sample sizes of the cohorts in our study also mitigate any potential variability across the cohorts.

Our study focused on genetic factors, though environmental and lifestyle factors, such as diet, physical activity, smoking, and socioeconomic status, were considered but not fully integrated, leading to potential residual confounding. Comorbidities and medication use, including hypertension, diabetes, and cardiovascular treatments, could independently influence HF risk. Interestingly, our study identified genetic markers that improved the risk prediction beyond those of conventional clinical variables, suggesting that our approach captures additional genetic contributors to HF risk. Environmental factors may contribute to the few cases amongst the low-risk subjects. Lastly, survivorship bias, where the controls included only individuals over 70 without HF, may underestimate genetic risk. Addressing these confounding variables through broader validation and clinical integration is essential for improving the test’s reliability.

HF subtypes and etiologies introduce genetic heterogeneity, limiting the predictive accuracy. HF is a heterogeneous condition with multiple etiologies, including myocardial ischemia, valvular heart disease, arrhythmias, and cardiomyopathy [27,28]. These subtypes have distinct pathophysiological mechanisms, which may influence the performance of our genetic test. While our two-phase approach addressed some aspects of genetic heterogeneity, the test’s predictive accuracy may vary across HF subtypes. For instance, Joseph et al. [28] recently reported differences in the genetic architecture of HF with a reduced ejection fraction (HFrEF) and HF with a preserved ejection fraction (HFpEF), with most loci associated with coronary disease and hypertension. Future research should explore the test’s performance in specific HF subtypes and integrate echocardiographic data to refine its predictive capabilities.

The implementation of a genetic test for HF risk in clinical practice raises important ethical and practical considerations. Communicating genetic risk to patients requires careful counseling to avoid misunderstandings and ensure informed decision-making. However, the ability to identify individuals at a high risk of HF before the onset of clinical symptoms offers significant opportunities for early intervention. Lifestyle modifications, such as weight management, BP control, and adherence to a heart-healthy diet, could delay or even prevent the onset of HF in genetically predisposed individuals

This study opens up several avenues for future research. First, the SNPs identified in our tests may point to novel molecular mechanisms underlying HF. Many of these SNPs are likely located in non-coding regions [29], regulating gene expression through mechanisms such as enhancer activity or chromatin remodeling. Investigating these mechanisms could provide new insights into HF pathogenesis and identify potential therapeutic targets. Second, expanding the test to include other ethnic groups and integrating environmental and lifestyle factors could enhance its predictive accuracy and clinical utility. Finally, longitudinal studies are needed to evaluate the test’s performance over time and assess its ability to monitor the disease progression and response to interventions [30].

Our findings align with recent genome-wide association studies (GWASs) and multi-trait analyses of HF, which have identified several significant loci associated with HF risk [31,32]. Eight of the SNPs in our test overlapped with the loci reported in these studies, supporting the validity of our approach. The lack of an overlap with other reported SNPs may result from differences in the study design, as we specifically searched for SNP-SNP interactive effects. Another explanation includes the different characteristics between study populations and the specific HF-related traits analyzed. This highlights the need for collaborative efforts to harmonize genetic data and refine risk prediction models.

## 4. Materials and Methods

### 4.1. Data from HF Cohorts

DNA and/or genetic and clinical data were obtained from three independent cohorts that differ significantly in their demographic and clinical characteristics. These cohorts are described as follows.

The Baker Biobank [33] enrolled individuals aged 18–69 years old between January 2000 and December 2011 and collected data on their socio-demographic characteristics, behavioural and lifestyle factors, anthropometric measurements, medical and medication history, and blood levels of various biomarkers (Supplementary Table S1). This study also collected and stored Guthrie cards, sera, plasma, buffy coat, and whole blood, collected in Tempus tubes (for RNA extraction). For some samples, extracted DNA and RNA was also stored. The BB data are also linked to echocardiogram, hospital admission, pathology, and mortality datasets. HF was defined according to medical records at the time of enrolment or using the ICD codes from linked admission and mortality datasets, as detailed previously [34]. DNA was extracted from all subjects with a diagnosis of HF, as well as controls aged over 70 without HF. DNA was genotyped using the Illumina GSA array (Illumina, San Diego, CA, USA) at the Australian Genome Research Facility (Parkville, Victoria, Australia). This produced genotypes of over 700,000 SNPs.

Atherosclerosis Risk in Communities: The ARIC study involved community-based surveillance, monitoring the incidence of myocardial infarction and coronary heart disease (CHD) and related mortality in a prospective cohort of 15,792 adults of different ethnicities, aged 45 to 64 years [35]. Its primary aims included identifying the determinants of subclinical atherosclerosis and CHD in middle-aged adults. Clinical and genetic data were obtained from the participating subjects after application to the National Institutes of Health dbGAP archive.

The Busselton Health Study: The BHS is one of the longest-running epidemiological research programs in the world [36]. It involves the residents of the town of Busselton in Western Australia, some of whom have been involved since 1966. Its primary focus is on cardiovascular and respiratory diseases and their risk factors. The BHS’s activities include a series of cross-sectional, whole-population health surveys, a follow-up of the survey participants, and the collection of sera and DNA samples. The genetic and clinical data of the participants of a survey from the 1990s were provided by the study’s co-ordinators after successful application.

### 4.2. Genetic Analyses

We employed three distinct methods to generate genetic signatures based on the disease data structures and distributed weights. We used a conventional association analysis, as well as searching for SNP-SNP interactions, as implemented in Plink 1.9 (http://pngu.mgh.harvard.edu/purcell/plink/) (accessed most recently on 14 February 2025) [37].

First, a case–control cohort study was designed, selecting as the controls individuals who were over 70 years of age without a diagnosis of HF from the BB cohort. This age threshold was used to minimize the inclusion of younger people in the training set, who may develop HF in the future. Other studies on the genetics of late-onset diseases have used a similar approach of excluding younger controls (e.g., [38]). We randomly divided the dataset into discovery (500 people) and testing (2167 people) subsets. Using the discovery subset, we implemented a multi-stage DNN pipeline to exploit extensive weight coverage effectively, incorporating weights from previous cycles. To ensure reliable results and exclude demographic noise, we restricted the training set to individuals of European descent within the discovery subset. A PCA was performed on such known individuals from other cohorts to verify their ancestry, and only individuals falling within the European-descent PCA range were included in the training set. Such individuals comprised 73% of the BB cohort. Analysing and interpreting these genomic data required high computational power and advanced data processing capabilities. Our tests were performed using the Pawsey Supercomputer Facility (www.pawsey.org.au) (accessed most recently on 16 February 2025). Our analysis culminated in the identification of 41 distinct SNPs, showing significant associations and interactions through the deep learning model, which successfully differentiated between the cases and controls.

We identified significant SNP-SNP interactions, which were incorporated into an artificial neural network. These interactions were included as weighted factors in the development of the QRS, enhancing the model’s predictive accuracy. Data from all subjects were included in a statistical model to comprehensively define risk, and a QRS was calculated for each subject in both tests. Subjects were then classified into high- or low-risk groups based on the QRS thresholds set at the 90th percentile of the control group.

A second-phase test was developed using input data only from the high-risk subjects, namely all true positive and false positive subjects identified in the first test. By masking the SNPs from Test 1, we found 29 SNPs that were used to define the risk for the second-phase test. Only whole-genome-typed genetic data were used for the development of both tests. After risk assignment using both Test 1 and Test 2, the tests were validated in a reserved “test set” of BB subjects whose data were not used for test derivation. Following this confirmation, the tests were further validated in the independent ARIC and BHS cohorts.

### 4.3. Genotype Imputation 

All SNPs used to characterize the BB cohort were directly genotyped. However, differences in reference panels and genotyping methods across cohorts led to high missingness in some of the 41 HF SNPs. In the ARIC and BHS cohorts, 8 SNPs in Test 1 and 5 SNPs in Test 2 had poor genotyping quality, resulting in missing data for 10% of individuals. To address these issues, genotype imputation was performed. We employed the Topmed Imputation Server, leveraging the updated Michigan Imputation Server Pipeline with Minimac4 [39]. We utilized the GRCh38hg38 data input. Older target genome builds for the BHS cohort were lifted over to align with GRCh38hg38. To evaluate the accuracy of the Minimac4 imputation, we blinded the original genotyped SNPs and compared them to the imputation results. This achieved over 99% accuracy, a significant improvement from the previous 68% accuracy using the 1000 Genome Panel Data in hg19.

### 4.4. Statistical Analyses

The statistical significance of the tests was evaluated using Fisher’s exact test, as implemented online (https://www.langsrud.com/fisher.htm) (accessed most recently on 16 February 2025). A two-sided *p*-value of less than 10E-5 was considered statistically significant. Other diagnostic scores, such as the OR and RR, were calculated using the standard methods (see ref. [40] for a review), with significance considered when the OR > 2 or the RR exceeded 1.5. The OR is a statistical measure that quantifies the association between an exposure (e.g., genetic risk) and an outcome (e.g., heart failure). An OR greater than 1 suggests increased odds of the outcome occurring with the exposure, while an OR of less than 1 indicates a protective effect. Both the ARIC and BHS cohorts are true community-based cohorts, making the use of the Relative Risk (RR) appropriate for their analysis. In contrast, BB is a case–control cohort, so the odds ratio (OR) was used to evaluate the test.

The performance of risk predictions can be visualized using KM survival curves, where the x-axis represents time factors, such as the patients’ age at diagnosis, and the y-axis indicates the proportion of disease-free individuals in the data. We generated KM survival curves for the different genetic risk groups using the Eureka Statistics online resource (https://eurekastatistics.com) (accessed most recently on 16 February 2025). This produces KM graphs and also calculates and displays the 95% confidence intervals for each group. The further apart the two curves on a KM survival graph are, the more significant the difference between the groups.

BAC, a performance metric used in classification problems, particularly when dealing with imbalanced datasets, was considered significant if it was greater than 0.6. It ensures a fair evaluation of the model’s performance by accounting for both the correct identification of positive cases and the correct identification of negative cases. It is calculated as the average sensitivity (true positive rate) and specificity (true negative rate). In our study, this metric provided a more comprehensive measure of our model’s performance across both classes.

To further understand how the genetic tests compared to the prediction of HF using standard clinical variables, we also performed survival analyses among the 1604 people without HF at baseline using multivariable Cox regression. From the BB cohort, 30 variables with previously described associations with HF were included in the model and discrimination assessed using C-statistics (similar to previous analyses [41]), with comparisons to Cox regression using the genetic test as the independent variable. No selection methods were used for variable inclusion given that the purpose was to maximize the model’s discrimination for comparison against the genetic test rather than to develop a parsimonious model that could be applied to other cohorts. Missing clinical variable data were managed using multiple imputation with chained equations (10 imputed datasets) using the “mice” package in R v4.2.2, with the results pooled using Rubin’s rules.

Adjusting for multiple comparisons to reduce the risk of Type I errors when multiple hypotheses are being tested simultaneously is an important aspect of a statistical analysis. We used Bonferroni correction to adjust for multiple comparisons to control the family-wise error rate (FWER), which refers to the probability of at least one false positive across all tests. This method was chosen because it is a conservative approach that reduces the likelihood of Type I errors across multiple statistical tests.

## 5. Conclusions

In summary, this study represents a significant step forward in understanding the genetics of HF. By developing a robust and nuanced genetic test for HF risk prediction, we have provided a tool with the potential to enable early intervention and personalized treatment strategies. While challenges remain, including the need for broader validation and the integration of non-genetic factors, our findings hold great promise for improving patient outcomes in HF, a condition that remains a significant cause of morbidity and mortality worldwide. Continued exploration and refinement of this test, along with its ethical implementation, will be critical to realizing its full potential in clinical practice. We welcome opportunities for collaboration in applying our genetic test to other populations.

## Figures and Tables

**Table 1:** Performance of the first-phase heart failure (HF) test on reserved Baker Biobank (BB) “test set” subjects. The results from this table were used to calculate the following statistics: *p*-value using Fisher’s exact test = 4.97 × 10^−25^; the Area Under the Curve (AUC) = 0.72; balanced accuracy (BAC) = 0.67; odds ratio (OR) = 2.53. The hazard ratio (HR) was calculated as 5.7.

Genetic Risk	Heart Failure Status	
	No HF	HF Cases
Low	523	313
High	530	801

**Table 2:** Performance of the second-phase HF test on the reserved validation set. The following statistics were calculated from these data: *p*-value using Fisher’s exact test = 8.1 × 10^−170^; AUC = 0.89; BAC = 0.81; OR = 50.29. The HR was calculated using the full test set, including controls under the age of 70, as 37.4.

Genetic Risk	Heart Failure Status	
	No HF	HF Cases
Low	412	52
High	118	749

**Table 3:** Performance of the combined HF test on the reserved “test set” subjects. Comparing the high- and low-risk subjects, *p* < 10^−300^, and the OR = 533.2. Comparing low- and intermediate-risk groups, *p* = 1.4 × 10^−57^, and the OR = 27.5.

Genetic Risk Group	Heart Failure Status	
	No HF	HF Cases
Low	603	12
Intermediate	365	200
High	85	902

**Table 4:** Validation of Test 1 in predicting HF risk in the independent Atherosclerosis Risk in Communities (ARIC) and the Busselton Health Study (BHS) population-based cohorts. Please note that these studies’ design means that cases comprise a much lower proportion than that in the BB case–control cohort. The data are shown for low- and high-genetic-risk groups, separated by HF status. One-sided *p*-values (*p*) from Fisher’s exact test and odds ratios (ORs) are presented for ready comparison with the BB results in Table 1. The CI (95% CIs) for the ORs further quantify the range of potential effect sizes, providing a measure of the uncertainty in the estimates. These calculations indicated very significant results, with a RR of 4.18 in the BHS cohort.

Cohort	Genetic Risk Group	Heart Failure Status		*p*	OR	95% CI
		No HF	HF Cases			
ARIC	Low	507	342	3.1 × 10^−8^	1.7	1.45–1.99
	High	498	562			
BHS	Low	1870	57	1.2 × 10^−30^	4.6	2.97–7.12
	High	1853	262			

**Table 5:** Validation of the combined HF tests in the ARIC and BHS cohorts. The data were stratified by genetic risk group (low- and high-risk) and HF status, with the one-sided *p*-values (*p*) and ORs demonstrating the improved predictive performance of the combined tests compared to that of Test 1 alone. The CIs (95% CIs) for the ORs indicate the precision of the estimates. The RR scores were 1.97 (ARIC) and 2.28 (BHS).

Cohort	Genetic Risk Group	Heart Failure Status		*p*	OR	95% CI
		No HF	HF Cases			
ARIC	Low	630	241	2.3 × 10^−57^	4.6	3.06–6.91
	High	375	663			
BHS	Low	2272	36	1.2 × 10^−70^	12.3	7.07–21.39
	High	1451	283			

## Data Availability

Restrictions apply to the availability of these data. Clinical and/or genetic data were obtained from the Baker Institute, the Busselton Health Study, and the National Institutes of Health. The data may be made available by these institutions on application. The predicted HF risk scores are available on request from the corresponding author following an embargo from the date of publication to allow for commercialization of the research findings.

## Supplementary Materials

The supporting information can be downloaded at https://www.mdpi.com/article/10.3390/ijms26041782/s1.
