# Supplementary material for: A Genetic Test to Identify People at High Risk of Heart Failure
Source: Int J Mol Sci. 2025 Feb 19;26(4):1782. doi: 10.3390/ijms26041782 (PMC11855781; doi:10.3390/ijms26041782)
Supplement: Supplementary file 1 [file ijms-26-01782-s001.zip › Table S3.pdf]

Pred	Controls	HF Cases
LR	416	125
HR	287	284

Supplementary Table S3. Performance of HF test on African-Americans reported in the ARIC cohort. The following statistics were calculated from these data: P-value by Fisher's exact test = 2.7e-28; Area under the Curve (AUC) =0.71; Balanced Accuracy (BAC) = 0.65; and Relative Risk = 2.15; The Hazard Ratio was calculated as 6.42.
