# Supplementary material for: A Genetic Test to Identify People at High Risk of Heart Failure
Source: Int J Mol Sci. 2025 Feb 19;26(4):1782. doi: 10.3390/ijms26041782 (PMC11855781; doi:10.3390/ijms26041782)
Supplement: Supplementary file 1 [file ijms-26-01782-s001.zip › Figure S1.pdf]

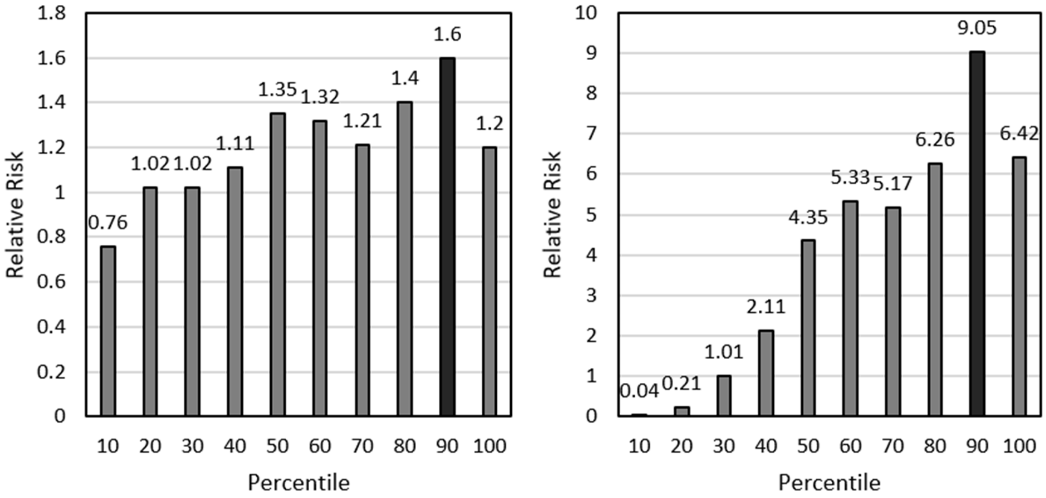

Supplementary Figure 1. Test performance of each decile. The histograms plot the Relative Risk against the Quantitative Risk Score (QRS) for Test 1 (left) and Test 2 (Right). We identified the optimal percentile of QRS values in the patient control group that gives the highest RR. Both Test 1 and Test 2 provided the best predictions at the 90th percentile of the controls.
