# Supplementary material for: A Genetic Test to Identify People at High Risk of Heart Failure
Source: Int J Mol Sci. 2025 Feb 19;26(4):1782. doi: 10.3390/ijms26041782 (PMC11855781; doi:10.3390/ijms26041782)
Supplement: Supplementary file 1 [file ijms-26-01782-s001.zip › Table S1.pdf]

Supplementary Table S1: Baker Biobank Characteristics

	Incident HF			
	No		Yes	
Patients	1051		1114	
Age	71.1		63.6	
Sex				
Female	416		321	
Male	635		793	
Body mass index	27.1		27.9	
Waist circumference	93.1		97.9	
Waist–hip ratio	0.91		0.94	
Smoking				
Never	585		430	0
Previous	46		92	1
Current	417		583	9
Alcohol (standard drinks/week)	2.98		2.41	
**Comorbidities**		*%*		*%*
Hypertension	640	*61%*	676	*61%*
Diabetes mellitus	123	*12%*	230	*21%*
(Insulin medications)	5	*0.5%*	12	*1%*
Coronary artery disease	347	*33%*	540	*48%*
Myocardial infarction	118	*11%*	785	*70%*
Valvular heart disease	39	*4%*	105	*9%*
Arrhythmia	202	*19%*	360	*32%*
Prior coronary artery bypass graft	143	*14%*	260	*23%*
Stroke	56	*5%*	502	*45%*
Chronic kidney disease	39	*4%*	100	*9%*
Asthma	135	*13%*	174	*16%*
Arthritis	129	*12%*	100	*9%*
Marfan's disease	1	*0%*	4	*0%*
Migraines	27	*3%*	31	*3%*
Retinopathy	99	*9%*	91	*8%*
Depression	35	*3%*	60	*5%*
**Metabolic profile**		*range*		*range*
Systolic blood pressure	140.0	*90-210)*	131.7	*(70-213)*
Diastolic blood pressure	78.7	*(25-125)*	76.4	*(40-130)*
Fasting glucose	5.9	*(1.1-23.3)*	6.5	*(2.0-24.7)*

Low-density lipoprotein cholesterol	2.9	*(0.4-6.4)*	2.7	*(0.5-7.7)*
High-density lipoprotein cholesterol	1.4	*(0.4-3.7)*	1.2	*(0.3-3.6)*
Triglycerides	1.6	*(0.4-11.2)*	1.9	*(0.5-10.6)*

Data represent n (%) for categorical data and median (minimum-maximum range) for continuous data.
