# Supplementary material for: A Genetic Test to Identify People at High Risk of Heart Failure
Source: Int J Mol Sci. 2025 Feb 19;26(4):1782. doi: 10.3390/ijms26041782 (PMC11855781; doi:10.3390/ijms26041782)
Supplement: Supplementary file 1 [file ijms-26-01782-s001.zip › Table S2.pdf]

Test	CHR	SNP	A1	F_A	F_U	A2	P	OR
1	1	rs10913019	G	0.1157	0.08773	A	2.59E-05	1.361
1	1	rs10797432	A	0.5146	0.4754	G	0.000434	1.17
1	1	rs2985714	A	0.118	0.09366	G	0.000332	1.294
2	1	rs946132	G	0.4057	0.4435	A	0.000589	0.8565
2	1	rs3935570	A	0.294	0.2587	C	0.000378	1.193
2	1	rs7552841	A	0.3426	0.3808	G	0.000365	0.8474
2	1	rs10494096	A	0.4416	0.4782	G	0.001086	0.863
2	2	rs1451826	A	0.1422	0.1735	G	0.000147	0.7896
1	3	rs11128793	A	0.3859	0.3423	C	4.44E-05	1.207
1	3	rs1231831	G	0.3369	0.3814	A	3.44E-05	0.8242
1	3	rs7618619	A	0.2795	0.3216	C	3.83E-05	0.8181
1	3	rs10511084	A	0.3248	0.3603	G	0.000807	0.8542
1	3	rs6769400	A	0.3988	0.4434	G	5.06E-05	0.8327
1	3	rs4507269	A	0.1924	0.2266	G	0.000172	0.813
1	3	rs2362965	A	0.4577	0.5068	T	1.13E-05	0.8213
1	3	rs17272530	A	0.5039	0.4675	G	0.001078	1.157
1	3	rs13097482	A	0.1515	0.1812	C	0.000362	0.8066
2	3	rs9834970	G	0.4455	0.5004	A	7.60E-07	0.802
1	4	rs309763	A	0.2064	0.1725	G	9.43E-05	1.247
1	4	rs10520496	G	0.3728	0.4095	A	0.000769	0.8574
1	4	rs6834781	G	0.4541	0.4146	A	0.000337	1.174
2	4	rs13150030	A	0.09905	0.07703	G	0.000408	1.317
1	5	rs447950	A	0.2747	0.3125	G	0.000207	0.8333
1	5	rs10073847	A	0.1523	0.126	G	0.00061	1.246
1	5	rs696836	C	0.1349	0.1088	A	0.000281	1.278
1	6	rs3130113	G	0.5009	0.5377	A	0.000918	0.8627
1	6	rs1954916	G	0.1671	0.1958	A	0.000864	0.8238
2	6	rs12943	G	0.257	0.2103	A	5.93E-07	1.299
1	7	rs13239964	A	0.1837	0.2157	G	0.000357	0.8184
1	7	rs9332401	G	0.1725	0.201	A	0.001078	0.8287
1	7	rs2312325	A	0.1899	0.1618	C	0.000829	1.215
2	7	rs854547	G	0.427	0.3882	A	0.000383	1.174
1	8	rs16899942	G	0.03823	0.02437	A	0.000252	1.592
1	8	rs11988552	A	0.03823	0.02382	G	0.000132	1.629
2	8	rs17120612	C	0.4303	0.3866	A	6.00E-05	1.199
2	8	rs4733601	G	0.4559	0.492	A	0.001175	0.865
1	9	rs34165419	A	0.08794	0.1116	G	0.000453	0.7677
1	10	rs11256439	C	0.2473	0.2804	A	0.000771	0.8431
1	10	rs4962613	A	0.4543	0.5087	G	9.81E-07	0.8039
1	10	rs11010872	G	0.2685	0.2372	A	0.001139	1.181
2	10	rs10509980	C	0.3877	0.3528	A	0.001143	1.161
2	10	rs1910534	A	0.2772	0.2442	G	0.000692	1.187
2	10	rs4751079	A	0.3317	0.3704	G	0.000267	0.8433
1	11	rs7952081	A	0.4363	0.4002	G	0.000977	1.16
2	11	rs7949406	G	0.4543	0.4992	A	5.33E-05	0.8352

1	12	rs11045232	A	0.04124	0.05956	G	0.000225	0.6792
2	12	rs17125266	A	0.03734	0.02199	G	2.97E-05	1.725
2	12	rs336264	G	0.1146	0.1403	A	0.000593	0.7928
1	13	rs3118913	G	0.1779	0.2072	A	0.000903	0.8281
1	13	rs11616892	A	0.2473	0.2794	G	0.0011	0.8474
2	13	rs3116599	G	0.1768	0.2058	A	0.001021	0.8288
1	14	rs712476	C	0.3319	0.2955	A	0.000434	1.184
1	14	rs8012291	A	0.119	0.1493	C	7.44E-05	0.7695
1	15	rs2573700	G	0.2279	0.2592	A	0.001089	0.8435
1	16	rs16968568	G	0.04982	0.03542	A	0.00115	1.428
1	16	rs1878090	A	0.4026	0.4476	G	4.78E-05	0.8319
1	17	rs9893995	A	0.02905	0.01743	G	0.00038	1.686
1	17	rs12946942	A	0.05786	0.07665	C	0.000876	0.7399
1	17	rs907089	G	0.3867	0.3487	A	0.000391	1.178
2	18	rs7407536	A	0.1669	0.1985	G	0.000261	0.8088
1	19	rs2562456	G	0.2149	0.2531	A	5.48E-05	0.8078
2	19	rs10084110	A	0.2919	0.2571	G	0.000429	1.191
1	20	rs2207418	G	0.2374	0.2725	A	0.000313	0.8311
1	21	rs11910142	G	0.2125	0.1801	A	0.000232	1.228
2	22	rs2294196	A	0.4184	0.4544	G	0.001129	0.8639

Supplementary Table S2: **Detailed information about the genetic variants**: This supplementary table provides information about the genetic variants used in the study. Each row in the table corresponds to a specific genetic variant, with key metrics as described in the following columns:

Test: The number of the genetic test performed.

CHR: The chromosome number where the single nucleotide polymorphism (SNP) is located.

SNP: The specific single nucleotide polymorphism being analyzed.

A1: The allele 1 variant of the SNP.

F_A: The frequency of allele 1 in the affected group (patients with heart failure).

F_U: The frequency of allele 1 in the unaffected group (controls without heart failure).

A2: The allele 2 variant of the SNP.

P: The p-value indicating the significance level of the association.

OR: The odds ratio representing the strength of the association between the SNP and the risk of heart failure.
